# Herpes Simplex Virus Establishment, Maintenance, and Reactivation: In Vitro Modeling of Latency

**DOI:** 10.3390/pathogens6030028

**Published:** 2017-06-23

**Authors:** Nikki M. Thellman, Steven J. Triezenberg

**Affiliations:** Van Andel Research Institute, Grand Rapids, MI 49503, USA; nikki.thellman@vai.org

**Keywords:** latent infection, lytic infection, HSV-1, HSV-2, neuron, quiescent infection, cell culture

## Abstract

All herpes viruses establish lifelong infections (latency) in their host, and herpes simplex viruses (HSVs) are highly prevalent worldwide. Recurrence of HSV infections contributes to significant disease burden in people and on rare occasion can be fatal. Cell culture models that recapitulate latent infection provide valuable insight on the host processes regulating viral establishment and maintenance of latency. More robust and rapid than infections in live animal studies, advancements in neuronal culture techniques have made the systematic analysis of viral reactivation mechanisms feasible. Only recently have human neuronal cell lines been available, but models in the natural host cell are a critical addition to the currently available models.

## 1. Introduction: Current Clinical Outlook

The significant global prevalence of herpes simplex viruses (HSVs) is largely due to the fact that a defining characteristic of the herpesviruses is lifelong infection. Recent models estimate that in 2012, the global prevalence for HSV type·1 (HSV-1) in people aged 0–49 years was 3.7 billion (67%) [1], and the prevalence for HSV-2 in people aged 15–49 years was over 400 million (11.3%) [2]. Herpesviruses employ two contrasting infection strategies to ensure both transmission of virus and long-term infection. Replication and viral shedding is referred to as lytic or productive infection, whereas latent infection refers to a quiescent state where viral genomic material persists in cells of the host, poised for reactivation.

HSV-2 is most commonly spread by sexual contact [3], whereas HSV-1 is frequently acquired through oral secretions as a mucosal infection during early childhood [4], although the epidemiology is changing such that many countries are seeing increasing sexually transmitted infections of HSV-1 [5]. Most often, the reactivation of latent HSV leads to recurring ulcerative blisters at the mucosal surfaces near the primary site of infection (orolabial or genital herpes) [6], but other painful lesions can occur as well [7]. Reactivation resulting in ocular lesions, such as herpetic epithelial or stromal keratitis, can lead to blindness [8,9]. In rare cases, HSVs can cause encephalitis [10], most notably in neonates born to mothers shedding either genital HSV-1 or HSV-2 [11,12] or in adults due to the reactivation of HSV-1 [13,14,15].

The lytic infection cycle of HSV has been well studied, and as such, is the primary target of most antiviral therapeutic drugs [16]. Acyclovir (acycloguanosine) was first designed as an antiherpetic drug in 1977 and remains the primary therapeutic approach today [17]. Acyclovir and its derivatives inhibit viral replication and reduce the duration and severity of clinical signs. Unfortunately, these therapies do not prevent recurrence arising from the reactivation of the latent virus [18]. In addition, viral strains resistant to acyclovir occur in immunocompromised patients [19,20,21,22] and in patients with herpetic keratitis treated with prophylactic acyclovir [23].

Therefore, new treatments that prevent the painful, sometimes life-threatening recurrence of HSV infections could benefit a significant portion of the human population. Unfortunately, the molecular mechanisms underlying viral reactivation are poorly understood, arising in part from a lack of robust experimental models in which the critical molecules involved in HSV reactivation can be studied. For many years, in vivo models using small animals (especially mice, rabbits, and guinea pigs) have been used to characterize latency in an anatomically and immunologically relevant context. Animal models are expensive and time-consuming. In vivo infections take over a month to establish latency. Moreover, latent virus is typically found in less than 20% of neurons [24,25], and only a small fraction of those latently-infected neurons will reactivate following experimental induction. As a result, molecular studies of viral reactivation mechanisms using in vivo models have been limited [26].

With recent advances in neuronal culturing techniques, however, in vitro cell culture models have emerged as powerful tools to study the molecular and genetic mechanisms of HSV latency and reactivation. Cell culture models offer several research advantages, including consistency, reproducibility, and cost-effectiveness. The expression or activity of viral and cellular proteins can be more readily modulated in cell culture by genetic approaches (including targeted gene editing), RNA interference, or pharmacological agents. In addition, confounding contributions from inflammatory or support cells are removed, providing an opportunity to study viral mechanisms at the single cell level. The purpose of this review is to examine the different HSV latency cell-culture models currently available, highlighting the specific contributions and advantages of each model.

## 2. HSV Life Cycle: Recapitulating the Latent Infection

HSVs are highly complex, with genomes that encode essential enzymes for gene regulation, DNA replication, processing, and packaging. In a lytic infection of the epithelium, virion protein 16 (VP16) is delivered as a tegument protein and drives viral gene expression by complexing with host cell proteins Oct-1 and HCF1. This VP16-induced complex recruits general transcription machinery to viral immediate early (IE) gene promoters, triggering lytic gene expression [27,28,29,30,31]. Lytic gene expression is highly ordered, with the IE gene products setting off a cascade of viral early and late gene expression, allowing DNA synthesis and nucleocapsid assembly to take place in the nucleus [32,33]. Virion assembly and maturation continues in the cytoplasm.

In contrast, latent infections are established under conditions in which the mechanisms driving the efficient, organized lytic life cycle are not adequately supported. Virus particles released from lytically-infected epithelial tissue enter the innervating sensory neuron axons. Capsids carrying viral genomes are released into the cytoplasm and traverse to the neuronal soma in ganglia driven by microtubule-based molecular motors; cytoplasmic dynein provides the motor force for retrograde movements towards the soma [34]. Capsids deliver viral genomes to neuronal nuclei where a non-productive infection (latency) occurs. One current hypothesis considers the architecture of the innervating neuron to be a key component in the process [35]. The extensive trafficking of capsids through long axons results in the inefficient transport of tegument proteins (such as VP16) to the nucleus, and subsequently in insufficient transcriptional activation of IE gene expression [35,36]. Without the robust expression of immediate early proteins, histone deposition across viral genomes is more prominent [37,38], favoring genome repression by epigenetic regulation.

Operationally, latency is described as “the persistence of a viral genome within tissue where, at any given time, there is a population of cells that lack detectable infectious virus, viral proteins, or viral lytic transcripts that are dormant but have the capability of being reactivated” [26]. During latency, the viral genome is stably maintained in the neuronal nucleus, where it persists as an episome [39] carrying marks of heterochromatin at lytic genes [40,41,42]. Viral genomes are therefore transcriptionally repressed during latency with the exception of expression of a non-coding RNA known as the latency-associated transcript (LAT). LAT is spliced to yield a stable 2.0 kb intron that accumulates in neurons and is believed to be important in maintaining HSV latency, although the mechanisms of LAT action remain under investigation [43,44]. Physiological triggers in the host result in cellular stresses that re-animate the latent virus in neurons; genomes are actively transcribed, replicate, and produce viral particles de novo that are transported back to the mucosal surface to cause a lesion (reactivation). The terminology used to describe HSV latency has been refined over the years, as recently reviewed by Sawtell and Thompson. They proposed a partitioning of the process of reactivation from latency into a series of events including pre-initiation (comprising reversible changes in signaling or viral chromatin), exit from latency (detectable expression of viral proteins), and reactivation (presence of infectious virus from cells where none had been present), and argue that consistent and common use of the latency lexicon will help to illuminate aspects of these mechanisms that might emerge from various experimental models [45].

The longstanding challenge in recapitulating HSV latency in cell culture involves simplifying a complex process with multiple steps (establishment, maintenance, and reactivation) [46] and multiple anatomical compartments (mucosa and ganglia). Consequently, most of the two-dimensional cell culture systems are permissive to lytic infection and require experimental conditions that favor non-productive infection, such as treatment with acyclovir, using low multiplicities of infection, or using replication-defective mutants. These experimental conditions do contribute to different limitations of different cell culture models. However, the analysis of molecular and genetic events specific to viral latency or reactivation is still more accessible in a cell culture compared to in vivo studies.

Defining the specific site of HSV latency, one aspect necessary in building a relevant model, has been historically challenging. The current consensus is that HSV is most frequently found in sensory neurons that innervate the primary site of infection [47]. For example, latent HSV-1 can be found in the trigeminal ganglia (TG) that innervate the lips, gingiva, and eyes, and latent HSV-2 can be found in the dorsal root ganglia (DRG) that innervate the skin and muscle of the back as well as the mucosa of the genitalia [48]. This consensus, however, is most likely an oversimplification; other ganglia, including sympathetic neurons from vestibular, geniculate, spiral, and sacral ganglia have been documented as sites of HSV latency [49,50,51,52]. Interestingly, an argument for non-neuronal sites of latency re-emerged in 2015, when evidence of HSV latency in corneas of patients with herpetic keratitis was reported [53]; to date, no robust model for ocular latency has yet been described. For now, many in the field agree that neurons are the preferred cell type in which to recapitulate latent infection in cell culture.

## 3. Latency Cell Culture Models

Unfortunately, reliable, ethical, and reproducible sources of neurons for cell culture investigation of HSV latency are hard to come by. For ethical reasons, human neuronal tissue is not readily available for primary culture. Here we examine the different HSV latency cell culture models that are currently available, which we segregate into three categories (i) non-neuronal; (ii) non-human neuronal; and (iii) human neuronal cell lines. Each model provides unique opportunities to study different aspects of HSV latency and reactivation (Table 1). In our opinion, when the findings from these models are taken into consideration collectively, progress in addressing critical gaps in understanding the molecular and genetic components of latency and reactivation can be made.

### 3.1. Non-neuronal Culture Models

Quiescent states that resemble HSV latency can be established in certain non-neuronal cell lines. Initially, HSV-1 quiescent infections were established in fibroblasts using a combination of elevated temperatures and replication inhibitors [54,55,56]. Quiescence with HSV-2 was established using elevated temperatures and a very low multiplicity of infection [57]. In another example, a non-replicating, non-toxic infection state was established in normal human diploid fibroblasts upon infection with HSV-1 mutants impaired for IE gene expression [58]. More recently, one report demonstrated quiescent wild-type HSV-1 infection of primary (serum starved) human diploid fibroblasts without the use of chemical replication inhibitors, by inducing heat shock (HS) proteins through elevated pre-incubation temperatures [59]. Using this fibroblast model and wildtype virus, entry into a quiescent state was characterized by the expression of some IE proteins (ICP4, ICP22), but a failure to process ICP22 and inefficient production of ICP0, all resulting in low-level viral replication [59]. These non-neuronal models have the advantage that the cells readily proliferate, generating ample material for experiments. Therefore, the pathways necessary for the establishment of latency can be studied in a human cell type in a rapid and robust manner.

Another interesting model uses rat pheochromocytoma (PC12) cells which were derived from a type of endocrine tumor from the adrenal gland that has neural crest origins [60]. Although not technically considered neurons, they can be differentiated into “neuron-like” cells when treated with nerve growth factor (NGF) or dexamethasone. Neuronally differentiated PC12 (ND-PC12) cells have been used to study HSV-1 quiescent infection from which the virus can reactivate following cellular stressors such as heat shock (43 °C for 3 h) or treatment with forskolin [61,62,63]. One specific study used a series of viral mutants with various deletions in the VP16 transactivation domain to evaluate the importance of specific sub-regions (VP16N and VP16C) for IE gene expression and stress-induced reactivation [64]. This approach is not feasible in vivo due to the poor replication of mutant strains. In ND-PC12 cells, however, equivalent viral genome copy numbers of mutant and wild-type viruses established quiescence. After reactivation was induced, neurons demonstrated unique regulation of IE gene activation and the VP16 transactivation domain was found to be critical for reactivation [64].

Both of these systems are simple and efficient; these cell lines proliferate readily and provide opportunities to use biochemical or molecular assays to study the quiescent infection of wild-type HSV-1. In addition, there is no need for the dissection of animal tissue for primary culture, thus saving time and reducing animal use. Diploid fibroblast models allow quiescent infection to be studied specifically in human cells, which provides a natural host-cell context. Unfortunately, fibroblasts are not the cell type in which latency and reactivation naturally occur [65]. Whereas ND-PC12 cells do offer a “neuronal-like” state, they are not human and only appear morphologically similar to neurons.

Host cell proteins that are critical in HSV gene regulation differ both in expression levels and cellular localization in neurons compared to non-neuronal cell types. For example, the POU (Pit-1/Oct/Unc-86) domain transcription factor Oct-1 is robustly expressed in epithelial cells and is, as previously mentioned, a critical gene regulatory protein that complexes with VP16 and HCF1 [32]. In sensory neurons, however, other POU domain proteins such as Brn-2 and Brn-3A are more robustly expressed [66] and these may bind to IE promoters with a higher affinity than Oct-1 [29,67]. In addition, HCF1 is an important cell cycle regulatory protein that in most cells localizes to the nucleus [68,69]. In sensory neurons, however, HCF1 localizes in the cytoplasm [70]. This may play a role in favoring a non-productive infection in neurons if HCF1 differentially binds VP16 in distal axons, impeding its trafficking to the nucleus [41]. Lastly, mouse fibroblasts lacking Oct-1 are impaired for HSV infection at low multiplicity but not high multiplicity, with deficits in both IE gene expression and DNA replication [71]. This supports the notion that mechanisms of HSV latency and reactivation should also be investigated in neurons, which presumably express the host cellular machinery of the natural site for viral latency.

### 3.2. Non-Human Neuronal Culture Models

The first latent infection of cultured neurons was reported by Wilcox and Johnson in the late 1980s, in which primary sympathetic neuronal tissue from prenatal rats was infected with either HSV-1 or HSV-2 in the presence of acyclovir [72,73]. Cultures that were initially shown to be quiescent (no viral antigens detected with immunohistochemistry) could be reactivated by NGF deprivation [73].

Some years later, the use of cultured rodent neurons re-emerged as a way to model latency in vitro. One group dissociated neonatal rat DRG and infected with lytic-gene reporter viruses developed on the backbone of a replication-defective mutant virus to better characterize primary infection and establishment of latency in neurons [74]. Specifically, this group demonstrated that from three days post-infection, the lytic phase expression was shut down whereas the expression from the LAT promoter was highly variable, suggesting that certain neuronal subtypes may be more permissive than others for LAT expression during latency [74]. The use of replication-defective virus strains limits the ability to study complete reactivation in this model, since productive infection is the final hallmark of reactivation. Still, early parts of the process of reactivation can be studied in this model; for example, NGF deprivation or treatment with trichostatin A (a histone deacetylase inhibitor) resulted in the activation of lytic promoters [74], giving an early indication for the role of epigenetic regulation in the maintenance of HSV latency.

Mohr and Wilson established latency using wild-type HSV-1 in a manner similar to the Wilcox model, in which superior cervical ganglia from prenatal rats were infected in the presence of acyclovir [75]. After treatment with an anti-mitotic agent, dissociated sympathetic neurons provided a near-homogenous population of cells that express one of the neurotrophin tyrosine receptor kinases, TrkA, which is expressed in some nociceptive sensory neurons. In this model, viral genomes were maintained at an average copy number of 25 per neuron, with LAT expression persisting in neuronal nuclei; lytic mRNAs and proteins and infectious virus were undetectable. Reactivation was induced by disruption of the NGF-TrkA pathway, either by direct NGF deprivation or by modulation of downstream signaling through PI3 kinase, AKT, and mTOR using shRNA knockdown or pharmacological inhibition [76,77], indicating that these signaling molecules were critical for maintaining the viral genome in a latent state [76]. Moreover, by growing primary neurons in compartmentalized culture systems such that media for axons are separated from media for neuronal cell bodies, spatial-specific parameters of HSV latency in neurons can be investigated [35]; latent HSV genomes in dissociated rodent ganglia soma responded to localized changes in mTOR signaling in axons [77]. The transition from latent to lytic transcription (reactivation) was carefully profiled using this model and revealed two distinct waves of viral mRNA accumulation during the exit from latency [41].

These models have enabled the molecular analysis of mechanisms necessary in maintaining HSV-1 in a latent state in a neuron, much like the cell type in the natural host. However, the permissiveness of rodent ganglia tissue to HSV lytic infection in culture means that either acyclovir treatment or replication-defective mutants are required to establish a non-productive infection. One potential critique of using acyclovir is that the guanine analogue may lead to stalled viral replication forks [78] and thus establish an aberrant latent state in which viral DNA is incompetent for subsequent replication and reactivation.

Some hypothesize that adult-rodent ganglia are less permissive to lytic infection than immature (embryonic or neonatal) ganglia and therefore do not require acyclovir. Margolis and Bertke infected dissociated adult mouse TG with HSV-1 and HSV-2 and demonstrated the establishment of latency without the use of acyclovir [79]. This adult sensory-neuron model also revealed a virus type (HSV-1 versus HSV-2) preference for establishing latency in specific nociceptive-neuron subtypes (A5+ versus KH10+, respectively) that may be driven by the differences in virus-type LAT expression [80,81]. Specifically, HSV-2 LAT contains a cis-acting regulatory element near the transcription start site that promotes productive infection in A5+ neurons and a second element in exon 1 that inhibits productive infection in KH10+ neurons, whereas HSV-1 does not contain such regulatory sequences and subsequently productive infection is not promoted in A5+ neurons [80].

A recent report illustrates the value of using non-human neurons to probe the roles of specific molecular pathways in regulating latency and reactivation. In this case, latent infection was established in dissociated sympathetic (superior cervical ganglia) and sensory neurons (TG) from adult mice using acyclovir [82]. The use of a number of pharmacological inhibitors revealed that during stress-induced reactivation, the c-Jun N-terminal kinase (JNK) activated the DLK/JIP-3 proteins which mediate the methylation and phosphorylation of specific amino acids of histone H3 in nucleosomes of latent viral genomes, and subsequently triggered early viral gene expression by initiating changes in chromatin structure that favor gene activation [82].

Compared to in vivo infections, latency can rapidly be established in dissociated primary ganglia (one week compared to one month) and results in an increased number of latently infected neurons, making the model more robust for molecular analysis. As such, these models are valuable for systematically dissecting the molecular details of latency establishment and maintenance, as well as the reactivation processes. In addition, a variety of ganglia types can be harvested and compared more easily than in vivo, which would require different infection sites. Comparing the efficiency of HSV latency in immature versus adult ganglia or sympathetic versus sensory ganglia, for example, may provide insight into specific neuronal molecular requirements for latency establishment, maintenance, or reactivation.

Nonetheless, rodent neurobiology is different than human neurobiology. The functional neuronal composition within anatomically distinct ganglia, for example, differs between species [83,84,85]. Therefore, specific molecular characteristics identified as important in rodent infection will need to be validated in human infections. One human autopsy study of TG was unable to identify a positive correlation of HSV-1 LAT expression in subtypes of nociceptive neurons [86], contradicting the notion (derived using animal models) of a viral preference for a specific subtype in latency establishment [80]. In addition, species differences exist in critical host molecules known to associate with viral proteins, with one example being Oct-1 [87]. As a result of these subtle differences in protein structure, VP16 has a lower affinity for rodent Oct-1 than for human Oct-1 [88]. As a component of the VP16-induced complex, Oct-1 is critical for driving IE gene expression, but this species-specific difference in protein affinity might confound findings involving viral molecular mechanisms and/or infection kinetics in rodents.

### 3.3. Human Neuronal Culture Models

Because access to primary human neuronal tissue is limited for ethical reasons, human neuron-like cell lines are an option to consider. Human neuroblastoma cell lines, such as SH-SY5Y cells, have been explored as infection models for HSV infection. SH-SY5Y cells were subcloned from a cell line isolated from a bone marrow biopsy of a child with neuroblastoma, and are therefore believed to be of neural crest in origin. In culture, proliferating SH-SY5Y cells can be differentiated from epithelial-like cells into a homogenous population of cells with a branched-neuronal phenotype [89,90]. Differentiated SH-SY5Y cells have a higher efficiency of HSV-1 uptake compared to undifferentiated (proliferating) SH-SY5Y cells [91]. Typically SH-SY5Y cells have been used in lytic infection studies or herpesviral vector studies [91,92,93]; the prospect that SH-SY5Y cells might be useful as a human neuronal HSV latency model has not yet been fulfilled [94]. Recently, one group demonstrated “dormant” HSV-2 infection in undifferentiated SH-SY5Y by using acyclovir [95], but as undifferentiated cells, SH-SY5Y lack a neuronal-like phenotype and a non-productive infection was not experimentally confirmed.

In a similar manner, a human neuronally-committed teratocarcinoma cell line (Ntera 2/D1, also known as NT2) can be induced by retinoic acid to differentiate into a neuronal-like phenotype designated hNT. Low-multiplicity infection by HSV-1 was impaired in such cells, and infection by ICP0-deficient viral strains resulted in a quiescent infection exhibiting some features of latency [96,97]. Given that replication-incompetent HSV vectors could persist for some time following infection of NT2 cells [97], they have been used for the further development of HSV-based viral vectors. However, little has been learned about the mechanisms of latency and reactivation in that setting.

Varicella zoster virus (VZV) is another human alphaherpesvirus that employs infection strategies similar to those of herpes simplex viruses. Without access to a neuronal cell line of human origin, the VZV field has taken advantage of the advancements in stem cell technology. Recently, a few infection models have been described based on human neurons differentiated from induced pluripotent stem cells (iPSCs) [98,99], neuronal stem cells [100], and embryonic stem cells [101,102]. Infection models using neurons from differentiated pluripotent cells are still in early stages, and as such major advances in HSV latency and reactivation specifically have not yet been made. In one study, approximately 80% of the cells derived after the differentiation of iPSCs were neuron-specific, but only 15% of the total cell population co-expressed sensory neuron markers [98]; using this model, undifferentiated iPSCs, neural precursor cells, and sensory neurons all supported lytic HSV-1 infection.

The process of neuronal differentiation results in a heterogeneous cell population wherein establishing latency may be challenging if permissive cells contribute to background virus production. For this reason, Gilden and colleagues began using iCells (Cellular Dynamics International), which are a mixture of post-mitotic neural subtypes derived from human iPSCs, in order to have a purer neuronal population in which to establish a latent, non-productive VZV infection [99]. To date, an HSV model has not been demonstrated in this relatively pure iPSC-derived cell line, but considering that iPSC-derived neurons are permissive to HSV-1 [98,103,104] infection, iCells may provide a promising human neuronal model in which to study HSV latency and reactivation. 

An alternative approach to acquire human neurons in culture arises from cell lines established from neuronal tissue. For example, the HD10.6 cell line was derived from human DRG and proliferates via a tetracycline-regulated v-*myc* oncogene [105]. In the presence of doxycycline, cellular proliferation is suppressed and cells mature to exhibit a sensory neuron-associated phenotype (SNAP cells) [105,106]. These cells were immortalized using the same technique as the Lund human mesencephalic (LUHMES) cell line, which was isolated from human mesencephalon and is widely used in neurodegenerative disease studies [107,108]. HD10.6 is the first available human cell line derived specifically from sensory neurons. The cell line can be rapidly expanded and eliminates the requirement for animal usage.

We obtained HD10.6 cells in order to develop a human in vitro latency model in which to study HSV reactivation. Following the precedent of models using dissociated rodent ganglia [75], a single treatment of acyclovir enabled a non-productive, quiescent infection in a small subpopulation of matured HD10.6 cells (SNAP cells) at relatively low viral genome copy numbers per cell [106]. HSV-1 maintained the capacity to reactivate in this model, but NGF deprivation induced only a modestly increased probability of reactivation relative to spontaneous reactivation [106]. SNAP cells therefore provide the opportunity to determine whether the neutrophin-signaling network responsible for maintaining latency in rodent ganglia is recapitulated in human neurons. In addition, the mechanism of LAT in HSV latency has never been studied directly in human sensory neurons and, at present, both LAT and lytic RNAs were repressed in this model. SNAP cells can be matured and infected in compartmented chambers and, like dissociated rodent ganglia [109,110], support axonal-only quiescent infections (unpublished data).

Human neuronal cell lines such as iCells or HD10.6 cells offer exciting platforms for studying the relevant molecular mechanisms of HSV latency in the natural host cell type. However, one caveat to consider is that, compared to in vivo rodent models, natural latent infection in human TG results in fewer neurons infected with HSV at lower genome copy numbers per cell [111]. Moreover, even in the face of external stressors, reactivation occurs infrequently in humans. Therefore, human-neuronal cell culture models may not prove to be as robust as rodent models and more advanced molecular techniques may be required.

## 4. Concluding Remarks and Future Directions

Advances in neuronal culturing techniques and the availability of high quality neuronal supplements have made it more feasible to study HSV latency and reactivation in dissociated ganglia from rodents. Technical approaches to culturing neurons, for example in fluidic chambers, have allowed sophisticated experimental designs in which viral and host mechanisms can be dissected based on spatial parameters more closely resembling natural infection. We have highlighted a variety of in vitro cell culture models that have been used to dissect critical viral and host mechanisms involved in the HSV latency cycle.

Studying latency mechanisms in neurons is ideal, but harvesting animal tissues is costly and tedious. Moreover, the field is aware of the caveats of using non-human models for human viral infections. Until recently, however, culturing human neurons for latent infection was not possible. Differentiation techniques have been fine-tuned such that relatively pure populations of neurons can be derived from human iPSCs or from commercial sources (iCells). An HSV latency model has yet to be demonstrated in iPSC-derived neurons and, although human, iPSC-derived neurons are not sensory-neuron specific. The HD10.6 cell line was derived from human dorsal root ganglia, and therefore more closely resembles sensory neurons.

It is intriguing to consider how latency mechanisms defined in the various cell culture models derived from different species will compare. Using these systems as complementary models to each other, various aspects of the molecular mechanisms of latency can be carefully dissected. Ultimately, the simplicity, reproducibility, and cost-effectiveness of these in vitro models will shed light on possible novel drug targets. Of course, with the simplification of cell culture comes the loss of critical contributing factors from the immunological or supporting environment in which a latently infected cell resides. The cancer field has demonstrated that contributions from surrounding non-tumor stroma and inflammatory cells (the microenvironment) can play significant roles in tumor progression, metastatic potential, and response to treatment. We predict that co-culturing of cells and organoid techniques will emerge as feasible tools for the analysis of HSV latency and will more accurately depict the physiology of latency while maintaining the benefits of an in vitro system.

## Figures and Tables

**Table 1:** Characteristics of cell culture models for HSV latency.

Model class	Example	Advantages	Disadvantages
Non-neuronal	Normal diploid human fibroblasts	Readily available, rapid proliferation, reproducible source	Not natural cell types for HSV latency
	Rat pheochromocytoma (PC12)	“	Host proteins important for virus infection may differ
Non-human neuronal	Rat prenatal sympathetic neurons	Suitable cell type for HSV latency	Rodent, not human; Molecular, physiological differences
	Rat neonatal or adult DRG, SCG neurons	“	Challenging to procure and culture
			No proliferation/long-term storage
Human neuronal	Neuroblastoma (SH-SY5Y), differentiated	Readily available, rapid proliferation, reproducible source	Neural crest but not neuronal origin: HSV latency not established
	Teratocarcinoma (NT2), differentiated	“	Not neuronal origin
	Induced pluripotent stem cells	“	Challenging to culture, heterogeneous , HSV latency not established
	iCells	Commercially available, less heterogeneous than iPSC-derived cultures	Expensive, HSV latency not yet established
	Immortalized ganglion cell lines (HD10.6)	Rapid proliferation, reproducible source, suitable cell type for HSV latency	Multiple neurotrophin receptors; inefficient reactivation of HSV
